# The Middlesex Hospital

**Published:** 1908-12-05

**Authors:** 


### THE MIDDLESEX HOSPITAL.

Major-General Lord Cheylesmore, speaking on November 26, at the Court of Governors of the Middlesex Hospital, said, in reviewing the work of the past six months, that the board profoundly regretted the death of the Earl of Derby, a true friend, who had been one of their members for seventeen or eighteen years, and came forward liberally in every time of need. It was intended to erect a memorial tablet to him in the chapel of the hospital, and the Secretary was prepared to receive subscriptions for that purpose. He also recorded with great sorrow the premature death of Sir Elliott Lees, a vice-president, who was a most generous benefactor to the wards of the Cancer Charity particularly. It was unanimously recommended that the Earl of Derby, himself, and the Hon. G. W. Spencer Lyttelton be appointed vice-presidents of the hospital, and that Dr. William Duncan and Mr. William Hern, retired from the active staff, be elected consulting obstetric physician and consulting dental surgeon respectively. Mr. W. Salmon Nowell had been appointed dental surgeon in succession to Mr. Hern, and Mr. H. Watson had been elected assistant dental surgeon. The new observation wards had proved to be of the greatest value during the past six months, and the scheme for the re-establishment of the lying-in ward had been successfully carried out, the beds having been ready and used since November 1. The trustees of the Zunz Bequest had given £2,500 towards the cost. He deeply regretted that their special appeal for funds for the Cancer Research Laboratories had not been so sucessful as they hoped it would be, and the board most earnestly urged that they should be helped in carrying on this great and beneficent work, for which they required £2,500 annually. Among the sums given were £100 each from Mr. Andrew Carnegie, Mr. E. S. Marcus, and Mr. Walter Emden (Scholarship), and the Drapers' Company had made a grant of £100 a year for five years and the Salters' Company a similar amount for three years to found a scholarship. To Mr. J. A. Mullens and his sons, who in a time of extreme financial difficulty had given a joint donation of £1,500 to the hospital, they gratefully acknowledged their indebtedness. The Chairman moved the adoption of a report setting out the details of Lord Cheylesmore's statement and sanctioning the sale of £1,040 2½ per cent. Annuities to meet the needs of the hospital. The resolution was adopted unanimously, and it was intimated that the board gave a cordial invitation to all interested to visit the hospital and see for themselves what is being achieved in the cancer wards and in the cancer research laboratories, the conviction being that only wider knowledge of the work is needed to create a keen sympathy with it and to bring more adequate financial support.

